# Docosahexaenoic Acid Increases the Pro-Resolving Brain Lipid Mediators of Inflammation in Rat Pups Prenatally Exposed to Alcohol

**DOI:** 10.3390/life15101530

**Published:** 2025-09-29

**Authors:** Enrique M. Ostrea, Deepak Yadav, Charlie T. Cheng, Esther D. Kisseih, Krishna R. Maddipati, Ronald L. Thomas

**Affiliations:** 1 Division of Neonatal-Perinatal Medicine, Department of Pediatrics, Hutzel Women’s Hospital and Children’s Hospital of Michigan, Wayne State University School of Medicine, 3901 Beaubien St., Detroit, MI 48201, USA; dyadav4@hfhs.org (D.Y.); charlietcheng@yahoo.com (C.T.C.); ekisseih@alumni.iu.edu (E.D.K.); thoma11r@cmich.edu (R.L.T.); 2 Bioactive Lipids Research Program, Wayne State University, Detroit, MI 48202, USA; maddipati@wayne.edu

**Keywords:** docosahexaenoic acid, alcoholic pregnant rats, newborn rat pups, inflammation, pro-inflammatory brain lipid mediator, pro-resolving brain lipid mediators

## Abstract

Fetal alcohol spectrum disorder (FASD/FAS) is a chronic inflammatory process of the fetal brain induced by alcohol and mediated by pro-inflammatory (PILM) and pro-resolving (PRLM) lipid mediators of inflammation. DHA (docosahexaenoic acid) is an essential precursor of PRLM. A study examining the response of lipid mediators of inflammation to alcohol insult and DHA supplementation can provide vital information on the pathogenesis of FASD/FAS and the potential ameliorative role of DHA. Four groups of timed pregnant rats were studied: control, low-dose (1.6 g/kg/day) and high-dose (2.4 g/kg/day) alcohol, and high-dose alcohol (2.4 g/kg/day) + DHA (1250 mg/kg/day). The pups were delivered on day 20, and their whole brain was examined for lipid mediators by liquid chromatography mass spectroscopy. The following biomarkers of brain lipid mediators were studied, namely, PILM (LTB4, PGE2, PGF2α, TXB2) and PRLM (LXA5, 4-HDoHE, 17-HDoHE, and MaR1n-3, DPA). The brain PILM and PRLM concentrations decreased significantly (*p* < 0.001) with high-dose alcohol. However, high-dose alcohol + DHA resulted in a significant (*p* < 0.001) increase in PRLM levels, viz., LXA5, MaR1n-3 DPA, 17-HDoHE, and a threefold increase in 4-HDoHE. We conclude that DHA supplementation in alcohol-exposed pregnant rats significantly increased levels of brain pro-resolving lipid mediators in the offspring, suggesting a potential role in modulating the inflammatory response.

## 1. Introduction

The body responds to noxious insults through inflammation, initially through the release of pro-inflammatory lipid mediators, such as prostaglandins and leukotrienes, followed by the release of pro-resolving mediators, e.g., resolvins, protectins, epoxins, etc. [1]. The precursor of the pro-inflammatory lipid mediators is Ω-6 arachidonic acid, whereas the Ω-3 polyunsaturated fatty acids, eicosapentaenoic acid (EPA) and docosahexaenoic acid (DHA), are the principal precursors of the pro-resolving lipid mediators [1].

Alcohol abuse among pregnant women is a serious health problem. In the United States, 1 in 7 pregnant women reported drinking alcohol within the past 30 days, and 1 in 20 engaged in binge drinking [2]. One of the serious complications of maternal alcoholism in the offspring is Fetal Alcohol Spectrum Disorder (FASD) and the Fetal Alcohol Syndrome (FAS) [3,4,5]. The pooled prevalence of FAS and FASD in the United States is approximately 2 per 1000 and 15 per 1000, respectively, in the general population [6,7].

The mechanism underlying FASD is still poorly understood, although unresolved inflammation, the generation of reactive oxygen species, and the lipid mediators of inflammation are important contributing factors [8,9,10,11]. The aim of our study was to assess, in a rat model, the pro-inflammatory and pro-resolving brain lipid mediators in response to chronic alcohol exposure at low (1.6 g/kg/day) and high (2.4 g/kg/day) alcohol doses and the effect of DHA supplementation in mitigating the inflammation process of alcohol at the high alcohol dose.

## 2. Materials and Methods

**Figure 1:**
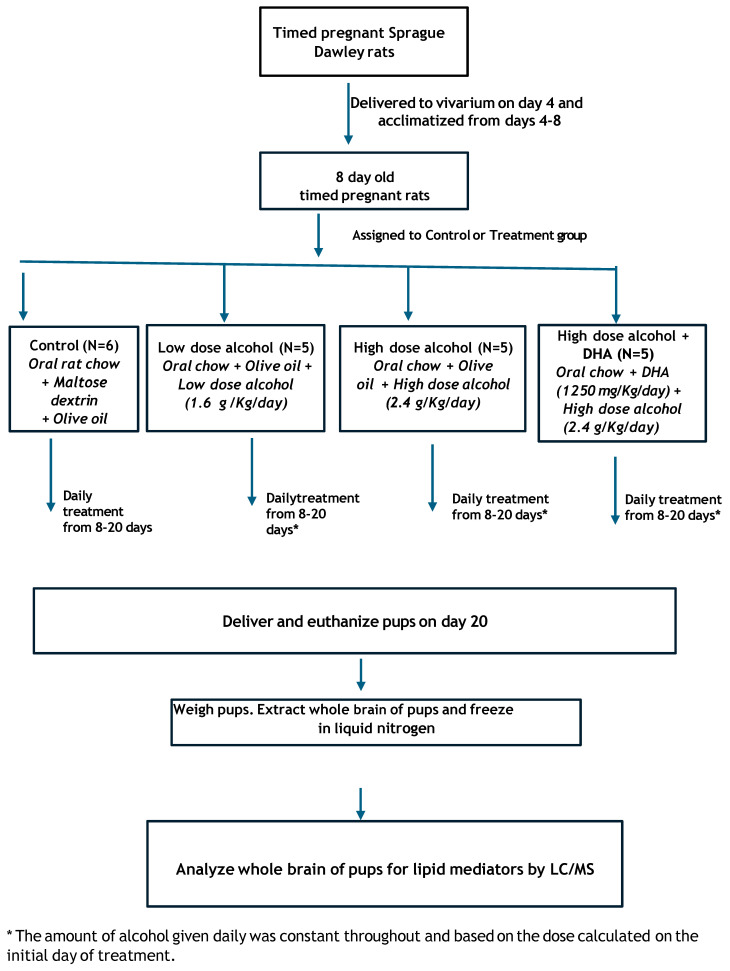
Schematic diagram of this study.

This study consisted of 4 groups of timed-pregnant Sprague-Dawley rats (dams)- See Figure 1.

Group 1. Control (C).

Group 2. Low-dose alcohol (LD) at 1.6 g alcohol/kg/day.

Group 3. High-dose alcohol (HD) at 2.4 g alcohol/kg/day.

Group 4. High-dose alcohol + DHA (HD/DHA) at 2.4 g alcohol/kg/day + DHA (1250 mg/kg/day).

The dams were delivered from the Charles River Laboratories to the vivarium on gestational day (GD) 4. Each dam was randomly assigned (Research Randomizer, www.randomizer.org) to either the control or treatment group. The animals were placed in individual cages and provided with standard rat chow and water ad libitum. The room was at a constant (22 °C) temperature on a 16 h dark/8 h light cycle (lights off from 5:30 p.m. to 9:30 a.m. the following day).

The study protocol was approved by the Institutional Animal Care and Use Committee (IACUC) of Wayne State University, Detroit, Michigan. (Protocol # IACUC-19-08-1222, Wayne State University).

### 2.1. Control Group (N = 6)

The control group was given daily gavage feedings in 2 divided doses of maltose/dextrin solution and olive oil starting from GD 8 to day 20. An equivolume of olive oil was given to the dams in groups 1–3 as a lipid control for the docosahexaenoic acid (DHA) given to group 4. Olive oil had a caloric load of 120 calories per 15 mL, and DHA had a caloric load of 123 calories per 15 mL. A maltose/dextrin solution served as the control for the caloric load of 2.4 g/kg/d of alcohol. Alcohol had a caloric load of 7 calories per gram, and maltose/dextrin had a caloric load of 95 calories per 25 g.

The total volume of maltose/dextrin/olive oil was approximately equal to the total volume of alcohol+olive oil given to the alcohol group and given by gavage in 2 divided doses, not to exceed the maximum safety limit of 10 mL/kg per gavage. Based on the initial weight of the dams, the volume of olive oil administered by gavage was 0.8 mL per day, and maltose dextrin was 1.8 mL per day.

Nine dams were initially assigned to the control group to anticipate the possible replacement of rats in the treatment groups that resisted initial gavage feedings. Three dams were subsequently reassigned to the treatment group, reducing the number of dams in the control group to 6. There was no pair-fed control group nor a group given alcohol alone, since our previous study showed that prenatal alcohol alone caused adverse effects of low birth and brain weights in the fetus, and low placental weight [12].

### 2.2. Alcohol Groups

A constant daily dose of alcohol was given to the treatment group based on the initial weight of the dam at the start of treatment (day 8). The study design was similar to our previous study [12] due to our concern that varying daily alcohol doses in the treatment group based on the changing weight of the dams would introduce an important confounder to the alcohol effect.

#### 2.2.1. Low-Dose (LD) Alcohol at 1.6 g Alcohol/kg/day (*N* = 5)

The stock solution of 29.85% (volume/volume) was prepared by adding 70 mL of distilled water to 30 mL of 99.5% ethanol solution (Spectrum Chemical MFG Corporation, New Brunswick, NJ, USA). At a density of alcohol of 0.7892 g/mL, the stock solution contained 0.7892 g/mL of alcohol. The low alcohol dose was 1.6 g/kg/d plus olive oil (volume for volume with DHA) given daily by gavage in 2 divided doses from gestational day 8 to day 20. Based on the different initial weights of the dams, the gavage volume of 29.85% alcohol that the dams received ranged between 1.6 and 1.9 mL per day and the volume of olive oil given was 0.8 mL per day.

#### 2.2.2. High-Dose (HD) Alcohol at 2.4 g Alcohol/kg/day (*N* = 5)

As with the low-dose alcohol group, a 29.85% stock solution of alcohol was used for the high alcohol dose of 2.4 g/kg/day, administered by gavage with olive oil (volume-to-volume with DHA) in two divided doses from gestational day 8 to day 20. Based on the initial weight of the dams, the gavage volume of 29.85% alcohol given to the dams in the alcohol + DHA group ranged from 2.2 to 2.5 mL per day. The volume of olive oil administered was 0.8 mL per day. We did not measure the initial blood alcohol level in the dams and estimated the alcohol level based on a similar study by Bielawski [13]. At an alcohol dose of 3 g/kg, administered in two divided doses to Sprague-Dawley rats, Bielawski et al. found the peak blood alcohol level in the rat at 0.5 h to be 53 mg/dL. In our study, the high-dose alcohol group was 2.4 g/day, which was below the 3 g/kg dose in the Bielawski paper, and we assumed that the BAC would be slightly below 53 mg/dL.

#### 2.2.3. High Dose Alcohol + DHA (HD/DHA) at 2.4 g Alcohol/kg/day + DHA at 1250 mg/kg/day (*N* = 5)

The DHA dose of 1250–2500 mg/kg was found to be safe and did not cause implantation losses, resorptions, abnormal sex ratios in live births, or fetal malformations [14]. DHA was obtained from DHASCO oil (DSM Nutritional Products, Columbia, MD, USA), which contains approximately 35% DHA (350 mg/mL DHA), 15% omega-6 docosapentaenoic acid (DPAn-6, 22:5n-6), and saturated and monounsaturated fatty acids. The full composition of the DHASCO oil is provided in Supplementary Materials S1. DHA was administered at a daily dose of 1250 mg/kg of body weight, along with 2.4 g/kg/day of alcohol, in two divided doses from gestational day 8 to day 20. Based on the initial weight of the dams, the gavage volume of 29.7% alcohol that the dams received in the alcohol + DHA group ranged from 2.2 to 2.5 mL per day. DHA was given at 0.8 mL per day.

#### 2.2.4. Gavage Feeding

Intubation of the dams for gavage feeding was initiated on gestational day 8 and continued until day 20. The feedings were given in two separate sessions, two hours apart, starting at 10 am. The gavage feedings were delivered through a curved, stainless steel, blunt needle (Perfectum #7916 CVD). No anesthesia or sedation of the dams was required, since the research members were well-trained in gavage insertion and feeding and the dams were gently and manually restrained with a dry towel.

The dams tolerated well the volume of ethanol plus DHA or olive oil that was administered at the high dose of 2.4 g alcohol/kg/day. Some dams were slightly intoxicated approximately 30 min after the alcohol feeding but regained motor ability, including normal feeding and drinking, within 4 h. They were monitored for 15 min after alcohol feeding to look for signs of labored breathing or distress, per the protocol of the Institutional Animal Care and Use Committee (IACUC) of Wayne State University.

#### 2.2.5. Delivery of the Dams and Pups

The usual gestational period for the dams is about 21 days, but we delivered the fetuses on GD 20 to prevent the spontaneously delivered pups on GD 21 from breastfeeding. On GD 20, the dams were quickly anesthetized with CO_2_ gas, and death was induced by puncturing the chest wall to cause a pneumothorax, opening the chest cavity, and cutting the heart. A laparotomy was immediately performed and the uterine horns exteriorized and opened to deliver the fetuses and the placenta. A total of 8 pups, closest to the uterus, were delivered per dam, four pups from each uterine horn. The pups were immediately weighed, decapitated, and their heads frozen in liquid nitrogen and stored at −80 °C for future analysis of brain lipid mediators of inflammation.

We could not determine the sex of the pups based on the standard measurement of anogenital distance, since the pups were very small and the key structures were difficult to identify. Thus, the offspring were not sex-stratified.

Since the brains of the pups were very small and soft, it was not possible obtain individual parts of the brain for separate analysis of lipid mediators. Instead, for each pup, we took the whole brain and analyzed it as one sample. However, we did not pool the brain of all the pups from each litter into one sample. Rather, we analyzed the whole brain of each pup separately.

#### 2.2.6. Brain Lipid Analysis

Thawed brain tissue from rat pups was used to prepare samples for liquid chromatography–mass spectrometry (LCMS) analysis. The pup’s head was retrieved from the deep freeze. The skull was opened to obtain the entire brain content, and the brain was weighed and thawed on ice. The thawed brain tissue was suspended in ice-cold phosphate-buffered saline (50 mM phosphate, 0.9% NaCl, pH 7.4) containing 10 mM butylated hydroxytoluene at a ratio of 1:9 (*w*/*v*, tissue to buffer). Zirconium beads were added to the sample and homogenized by high-frequency oscillation (PreCellys, Bertin Instruments, Rockville, MD, USA). The homogenate was centrifuged at 1000× *g* for 10 min and the supernatant was collected for lipid mediator extraction and analysis as previously reported [15,16]. Briefly, the homogenate was supplemented with 5 ng each of stable isotope-labeled internal standards (Prostaglandin E1-d4, Resolvin D2-d5, Leukotriene B4-d4, 15-hydroxyeicosatetraenoic acid-d18 (HETE), and 14R,15S-epoxyeicosatrienoic acid (14,15-EpEtrE-d11)—Cayman Chemicals, Ann Arbor, MI, USA) and applied to StrataX Solid Phase Extraction columns (Phenomenex, Torrance, CA, USA). The eluates were analyzed by LCMS on a QTRAP5500 mass spectrometer following high-performance liquid chromatography (HPLC) separation on a Luna C18(2) (2 × 150 mm, 3µ) column as previously described [15,16]. Lipid mediator concentrations were calculated against the internal standards and normalized to the protein concentration in the homogenate (ng/mg protein).

#### 2.2.7. Statistical Analysis

We enrolled eight pups from the litter of each dam consisting of four pups from each uterine horn. The sample size provided us with 90–95% power to detect a medium effect size among groups and at *p* < 0.05 for statistical significance. Each lipid metabolite was an independent measure and was compared to the type of treatment for groupwise changes, since the aim of this study was to compare specific lipid metabolite concentrations based on treatment groups and not between metabolites. Homogeneity of variance was assessed using Levene’s test; if variances were equal, the means were compared using one-way analysis of variance (ANOVA). If variances were unequal, the non-parametric Kruskal–Wallis (H test) was used for pairwise comparisons. *p* < 0.05 in either the ANOVA or Kruskal–Wallis tests was considered as the level of statistical significance.

An unadjusted Dunn’s test was used to compare the dependent variable across each pairing of the independent variable to examine possible significant differences. To reduce the risk of Type I errors (or false positives), a Bonferroni correction was applied to adjust the *p* values.

**Figure 2:**
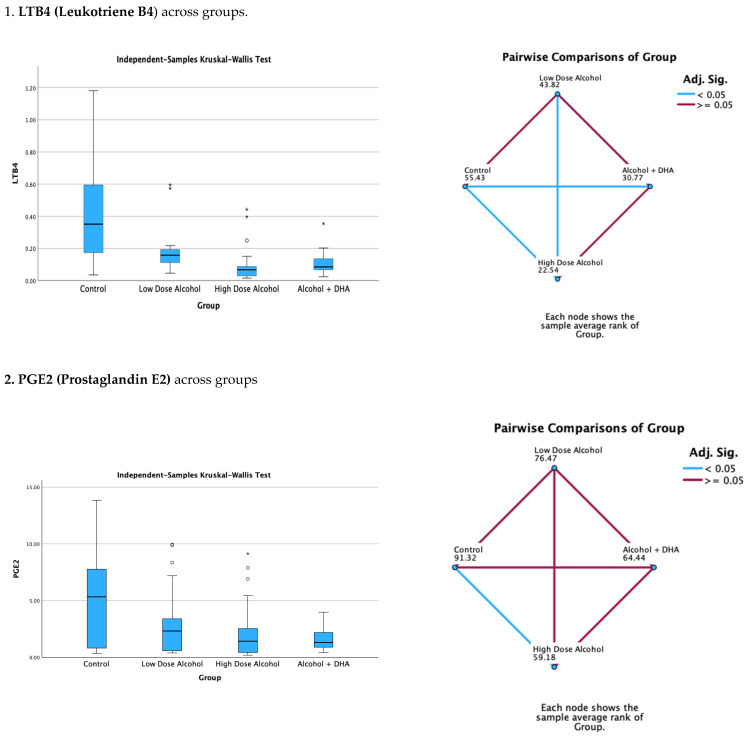

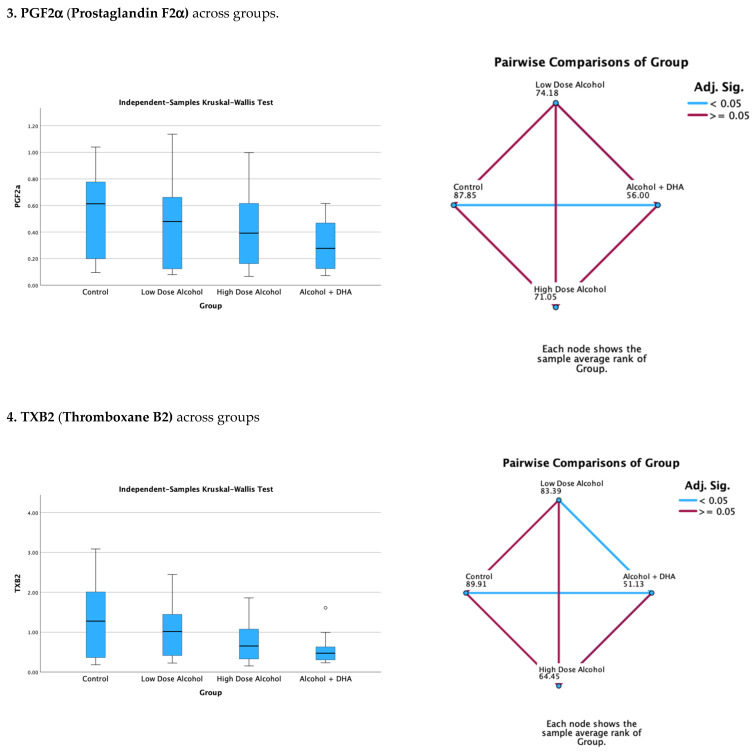
The effect of alcohol with or without DHA on the pro-inflammatory lipid mediators of inflammation, viz., LTB4 (Leukotriene B4), PGE2 (Prostaglandin E2), PGF2a (Prostaglandin F2α), and TXB2 (Thromboxane B2), in the fetal rat brain. All data are in ng/mg protein of pup brain homogenates and presented as median and interquartile range. Asterisks in bar graphs stand for extreme values.

**Figure 3:**
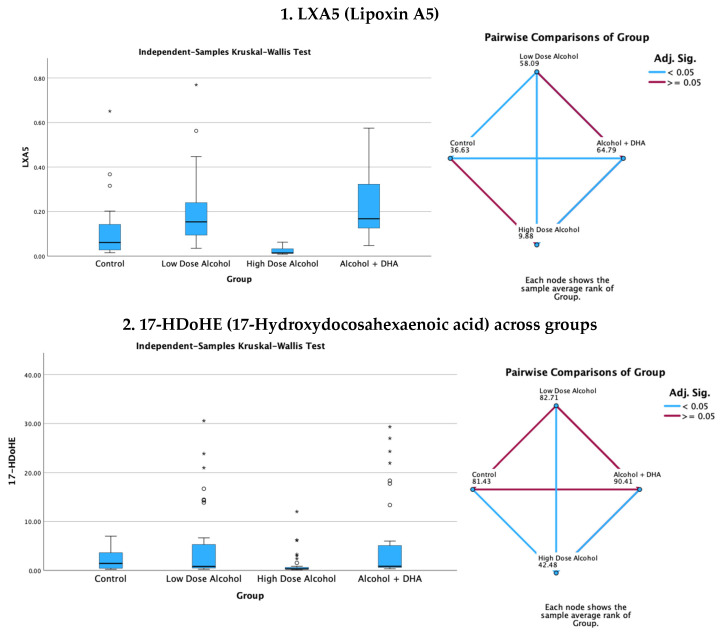

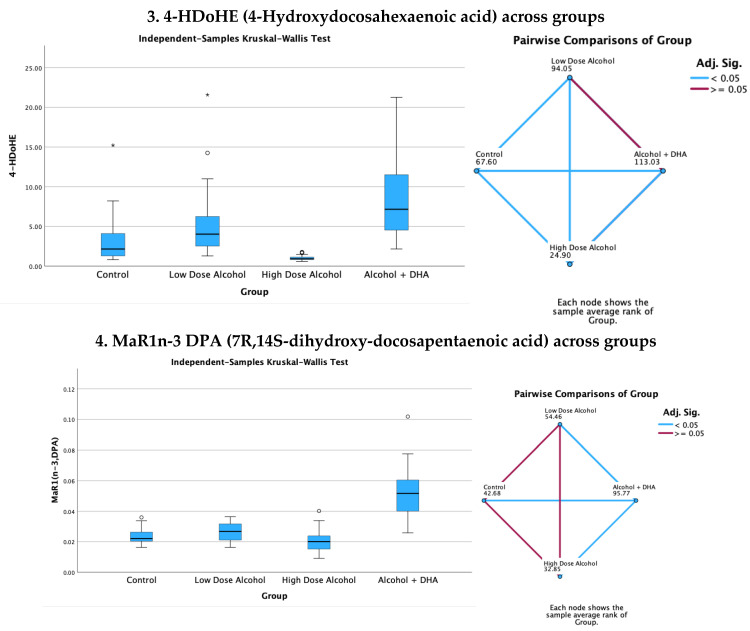
Effect of alcohol with or without DHA on the pro-resolving lipid mediators, e.g., LXA5 (Lipoxin A5), 17-HDoHE (17-Hydroxydocosahexaenoic acid), 4-HDoHE (4-Hydroxydocosahexaenoic acid), and MaR1n-3 DPA (7R,14S-dihydroxy-docosapentaenoic acid), in the fetal rat brain. All data are in ng/mg protein of pup brain homogenates and presented as the median and interquartile range. Paired comparisons and “*p*” values were analyzed by the Kruskal–Wallis test with the red line in the figure indicating no significant relationship at *p* > 0.05 and the blue line indicating a significant relationship at *p* < 0.05.

Levene’s test was used to assess the equality of variances across different groups. The test evaluates whether the variances in different samples are significantly different from each other. This is particularly important because many statistical tests assume that the variances of the populations from which the samples are drawn are equal. Given the number of groups examined in the model, we chose Levene’s test as an appropriate selection. Although the Welch’s ANOVA is generally more powerful than the Kruskal–Wallis test under specific conditions related to data characteristics, the Welch’s ANOVA is more powerful than the Kruskal–Wallis test when the data is normally distributed, variances are unequal, sample sizes are large, and effect sizes are significant. The graphs shown in Figure 2 and Figure 3 display positively skewed distributions, and since the sample sizes used in our study within each group were small, we interpreted these as sufficient evidence to perform the non-parametric Kruskal–Wallis test.

Statistical analysis was performed using SPSS version 29.

## 3. Results

### 3.1. Birth Weight

The mean birth weight of the pups when delivered on day 20 of gestation is shown in Table 1 [17].

The number of fetuses reported is the number of fetuses obtained per litter and not the litter size. The pups in the high-dose alcohol group had a significantly higher mean birth weight compared to the control group (4.10 g vs. 3.06 g, *p* <0.05). Similarly, the pups in the high-dose alcohol/DHA group had a significantly higher birth weight compared to the control group (3.54 g vs. 3.06 g, *p* = 0.05). The pups in the high-dose alcohol group had a significantly higher birth weight than the pups in the high-dose alcohol/DHA group (4.10 g vs. 3.54 g, *p* = 0.02). There was a significant difference in the birth weight between the pups in the LD alcohol vs. HD alcohol group (3.08 g vs. 4.10 g, *p* = 0.05) and the LD vs. HD alcohol/DHA group (3.08 g vs. 3.54 g, *p* = 0.05). There was no significant difference in the mean birth weight of pups in the LD alcohol vs. control group (3.08 vs. 3.06, *p* > 0.05).

### 3.2. Dam Weights

The mean weight of the dams on gestation days 8 and 20 are shown in Table 2 [17]. There was no significant difference in the dam weight in the different treatment groups on GDs 8 and 20. The alcohol dose was calculated from the initial weight of the dams.

### 3.3. Placental Weights

The mean placental weights from each treatment group are shown in Table 3 and Table 4 [17]. The mean placental weight in the control group was significantly smaller in the control group compared to the treatment groups (ANOVA, *p* < 0.001).

### 3.4. Brain Lipid Mediators of Inflammation

Many cyclooxygenase- and lipoxygenase-derived pro-inflammatory and pro-resolving lipid mediators were analyzed in the pups’ brains in response to alcohol dose and DHA supplementation of the dams. The different brain lipid mediators were compared based on their treatment group, and only those mediators with a significant difference (*p* < 0.05) when comparing group treatments are presented in this report. These brain lipid mediators included 1) the pro-inflammatory lipid mediators (PILMs), e.g., leukotriene B4 (LTB4), prostaglandin E2 (PGE2), prostaglandin F2 (PGF2), and thromboxane B2 (TXB2); 2) the pro-resolving lipid mediators (PRLMs), e.g., lipoxin A5 (LXA5), 4-Hydroxydocosahexaenoic acid (4-HDoHE), 17-Hydroxydocosahexaenoic acid (17-HDoHE), and 7R,14S-dihydroxy-docosapentaenoic acid (MaR1n-3 DPA).

The pro-inflammatory lipid mediators are derived from the cyclooxygenase pathway (PGE2, PGF2alpha, and TxB2) and the lipoxygenase pathway (LTB4). The pro-resolving mediators (LXA4, 4 and 17 HDoHE, and MaR1) originate from the lipoxygenase pathway.

The mean concentrations of the brain lipid mediators were significantly skewed, and their variances were non-homogeneous. Therefore, the Kruskal–Wallis H test was used to compare the groups based on treatment.

The results of the Kruskal–Wallis H test comparing median brain lipid mediator concentrations across treatment groups are shown in Figure 2 and Figure 3. There were three degrees of freedom.

(Note: DHASCO, which was the primary source of DHA in this study, contains other polyunsaturated fatty acids, as shown in Supplementary Materials S1. Therefore, the possible contribution of these other fatty acids cannot be excluded from the beneficial effects attributed to DHA alone. Although the DHA dose given to the dams was based on its actual content in DHASCO, the term “DHA” in the study refers to the combined effect of DHA and other polyunsaturated fatty acids present in DHASCO.)

#### 3.4.1. Pro-Inflammatory Brain Lipid Mediators

The effects of alcohol with or without DHA on pro-inflammatory lipid mediators, such as LTB4 (Leukotriene B4), PGE2 (Prostaglandin E2), PGF2a (Prostaglandin F2), and TXB2 (Thromboxane B2), in the fetal rat brain are shown in Figure 2. All data are expressed in ng/mg protein of pup brain homogenates and are presented as the median with interquartile range.

Paired comparisons and p-values were analyzed using the Kruskal–Wallis test, with the red line in the figure indicating no significant relationship at *p* > 0.05 and the blue line indicating a significant relationship at *p* < 0.05.

- Legend: Control = olive oil + maltose/dextrin;
- Low-dose alcohol = 1.6 g/kg/day alcohol + olive oil;
- High-dose alcohol = 2.4 g/kg/day alcohol + olive oil;
- Alcohol + DHA = alcohol (2.4 g/kg/day) + DHA (1250 mg/kg).

Summary of paired comparisons of pro-inflammatory brain lipid mediators based on treatment:

1. The low-dose alcohol group was not significantly lower than the control in the four pro-inflammatory lipid mediators.
2. The high-dose alcohol group was significantly lower than the control in LTB4 and PGE2 (Figure 2).
3. The high-dose alcohol + DHA group was significantly lower than the control in LTB4, PGF2α, and TXB2.
4. High-dose alcohol was significantly lower than low-dose alcohol in LTB4.
5. High-dose alcohol + DHA was not significantly different compared to high-dose alcohol in any of the pro-inflammatory lipid mediators.

#### 3.4.2. Pro-Resolving Brain Lipid Mediators

The effect of alcohol, with or without DHA, on the pro-resolving lipid mediators, e.g., LXA5 (Lipoxin A5), 17-HDoHE (17-Hydroxydocosahexaenoic acid), 4-HDoHE (4-Hydroxydocosahexaenoic acid), and MaR1n-3 DPA (7R,14S-dihydroxy-docosapentaenoic acid), in the fetal rat brain are shown in Figure 3. All data are expressed in ng/mg protein of pup brain homogenates and are presented as medians with interquartile range.

Paired comparisons and “*p*” values were analyzed using the Kruskal–Wallis test, with the red line in the figure indicating no significant relationship at *p* > 0.05 and the blue line indicating a significant relationship at *p* < 0.05.

- Legend: control = olive oil + maltose/dextrin;
- Low-dose alcohol = 1.6 g/kg/day alcohol + olive oil;
- High-dose alcohol = 2.4 g/kg/day alcohol + olive oil;
- Alcohol + DHA = alcohol (2.4 g/kg/day) + DHA (1250 mg/kg).

Summary of paired comparisons of pro-resolving brain lipid mediators based on treatment:

1. The low-dose alcohol group was significantly higher than the control in LAX5 and 4-HDoHE.
2. The high-dose alcohol group was significantly lower than the control in 4-HDoHE and 17-HDoHE. Alcohol + DHA was significantly higher than the control in LXA5, MaR1n-3 DPA, and 4-HDoHE.
3. High-dose alcohol was significantly lower than low-dose alcohol in LXA5, 4-HDoHE, and 17-HDoHE and vs. the control in 4-HDoHE and 17-HDoHE.
4. High-dose alcohol combined with DHA, compared to high-dose alcohol alone, showed a significant increase in LXA5 and MaR1 (n-3 DPA), and a two- to three-fold rise in 4-HDoHE (7.163 vs. 2.155 ng/mg, *p* < 0.001) and 17-HDoHE levels (0.850 vs. 0.317 ng/mg, *p* < 0.001), despite the high alcohol dose.

## 4. Discussion

Inflammation is a major protective response of the body to noxious insults [1]. The initial inflammatory response involves specific cellular infiltrates, such as neutrophils, and the release of pro-inflammatory lipid mediators, including prostaglandins, cytokines, and leukotrienes [1]. This is soon followed by the cessation of PMN influx, the clearance of debris by macrophages, and the production of pro-resolving lipid mediators known as specialized pro-resolving mediators (SPMs), such as lipoxins, resolvins, protectins, and maresins, which evoke potent anti-inflammatory and pro-resolving responses as well as enhance microbial clearance [1]. SPMs include ω-6 arachidonic acid-derived lipoxins, ω-3 eicosapentaenoic acid (EPA) and docosahexaenoic acid (DHA)-derived resolvins, protectins and maresins, cysteinyl-SPMs, and n-3 docosapentaenoic acid (DPA)-derived SPMs [1,18].

In a rat model, our study found many of the cyclooxygenase and lipoxygenase-derived pro-inflammatory and pro-resolving lipid mediators in the brain of rat pups chronically exposed to alcohol and DHA during gestation. These included pro-inflammatory lipid mediators such as LTB4, PGE2, PGF2α, and TXB2; pro-resolving mediators such as lipoxin LXA5 and MaR1n-3DPA; and resolvin precursors such as 4-HDoHE and 17-HDoHE (Figure 2 and Figure 3).

The study showed no significant increase in pro-inflammatory lipid mediators in the low-dose alcohol group (1.6 g/kg/day) compared to the control, likely due to the low alcohol dose and the potential beneficial effect of olive oil. Conversely, high-dose alcohol (2.4 g/kg/day) without DHA supplementation resulted in a significant (*p* < 0.005) reduction in pro-inflammatory lipid mediators, such as LTB4, PGE2, and TXB2, as well as a significant decrease in pro-resolving lipid mediators such as LXB4, 4-HDoHE, and 17-HDoHE.

Although the expectation was that both pro-inflammatory and pro-resolving brain lipid mediators would increase in response to alcohol exposure, this was not observed, likely due to significant neuronal degeneration and death caused by prolonged alcohol toxicity. Alcohol has been shown to induce apoptotic neuronal degeneration and death through the activation of caspase 3 and 9 [19,20], DNA fragmentation, nuclear disruption [21], oxidative stress that coincides with the induction of pro-inflammatory cytokines TNF-α, IL-1β and TGF-β [22], enhanced acetylcholinesterase activity, and increased oxidative-nitrosative stress [22,23].

Although a high dose of alcohol alone caused a significant decrease in both pro-inflammatory and pro-resolving lipid mediators in the pups’ brains, the opposite was observed with alcohol + DHA supplementation. Despite the high alcohol dose, many pro-resolving brain lipid mediator levels were significantly increased with high alcohol combined with DHA. These included (1) a significant rise in LXA5 and MaR1n-3DPA, (2) a threefold increase in 4-HDoHE, and (3) an increase in 7-HDoHE concentration (Figure 3). Therefore, DHA’s ability to increase pro-resolving brain lipid mediators provides strong evidence of its protective role in counteracting the harmful effects of high alcohol intake on the fetal brain.

Resolvin precursors were enhanced by DHA supplementation in the alcohol-exposed fetal pups. Resolvin D-series are produced from the oxidation of DHA by 15-lipooxygenase to 17-hydroperoxydocosahexaenoic acid and metabolized further into resolvin Ds. The resolvin E series are produced from EPA by acetylated cyclooxegenase-2 or cytochrome P450 [24]. Resolvin was discovered by Serhan and his team in their search for potential endogenous bioactive compounds derived from omega-3 fatty acids [25]. The pro-resolving and anti-inflammatory effects of resolvins are predominately achieved through specific G-protein-coupled receptors [26]. Resolvins can inhibit microglia activation and reduce the pro-inflammatory cytokines, such as TNF-α, IL-6, IL-1β, iNOS, and nitric oxide, through the MAPK, NF-κB, PI3K/Akt, and caspase-3 signaling pathways [25]. The many actions of resolvin in resolving inflammation-mediated diseases are extensively discussed in several review articles [23,24,25,26,27].

In a previously published report [17], we measured cytokine levels in the dams, which showed higher pro-inflammatory cytokines (principally interleukin-1β, interleukin-12p70, and TNF-α) in the alcohol-exposed pregnant rats compared to the control. However, the difference was not statistically significant, due to the large variance in the mean concentrations and the small sample size. Nonetheless, the literature shows an increase in brain cytokines from alcohol insult secondary to microglia activation [28] and an increase in the brain cytokines, TNF-α, IL-1β, and TGF-β in alcohol-exposed rat pups [21].

DHA is one of the important long-chain polyunsaturated fatty acids (LCPUFAs) in the body. It is a major component of the pro-resolving lipid mediators of inflammation [18] and an essential element in the growth and development of the brain and retinal development in infants and normal brain function in adults [28,29]. DHA deficiencies are associated with Fetal Alcohol Syndrome, attention deficit hyperactivity disorder, cystic fibrosis, phenylketonuria, unipolar depression, aggressive hostility, and adrenoleukodystrophy [30].

Deficiency in DHA can occur in the fetus secondary to several conditions. Prematurity is a significant factor since the premature infant cannot sufficiently synthesize DHA, and a maternal source is necessary [31]. LCPUFA is increasingly transferred from the mother to fetus late in pregnancy, reaching peak accretion rates between 42 and 75 mg/d at 35–40 weeks of gestational age [32,33]. Thus, premature delivery of the infant results in lower levels of DHA and other vital PUFAs due to the insufficient transfer of these fatty acids from the mother. In maternal alcohol disorder, the deficiency of DHA in the fetus is further aggravated since alcohol depletes DHA by the esterification of DHA into fatty acid ethyl esters (FAEEs), which is excreted in the urine [34]. DHA deficiency has been observed in the plasma levels of women with high alcohol intake during pregnancy and, consequently, a low placental transport of DHA to the fetus [35].

Prenatal alcohol exposure in the developing rat brain appears to decrease PUFA concentrations of membrane phospholipids [36,37], and the deficiency of DHA in the fetus has been shown to result in poor cognitive and behavioral performance [33,38,39,40,41]. Supplementation of pregnant women in their diet with n-3 PUFA has appeared to have increased DHA levels, reduced placental oxidative stress, and enhanced placental and fetal growth [42,43]. The postnatal omega-3 supplementation of pups exposed to prenatal alcohol also partially reduces oxidative stress and reverses long-term deficits in hippocampal synaptic plasticity [44,45].

Although our primary aim in this study was to determine the salutary role of DHA on brain lipid mediators of inflammation in rat pups prenatally exposed to alcohol with olive oil as the lipid control for DHA, there was an unexpected secondary finding of a significant increase in birth weight with olive oil, even in the alcohol-exposed pups [17]. The rise in birth weight could have resulted from the calories provided by alcohol and olive oil, since olive oil has 8.5 calories per gram and alcohol has 7 calories per gram. Likewise, the positive effect of olive oil on birth weight was more noticeable in the high-dose compared to low-dose alcohol groups, probably due to the higher caloric intake in the high-dose alcohol group. The pups’ birth weight was also higher in the high-dose alcohol/olive oil group compared to the high-dose alcohol/DHA group, possibly because of DHA loss from esterification with alcohol [34]. It is also possible that increased visceral fat biosynthesis and accumulation, due to the interaction of alcohol and the high saturated fat content in olive oil, contributed to these results [46].

Olive oil contains hydroxytyrosol (HT), a key polyphenol with anti-inflammatory and neuroprotective effects. Hydroxytyrosol is quickly absorbed and demonstrates cytoprotective properties by scavenging free radicals and reducing inflammation [47,48]. In animal studies, maternal HT supplementation has also been associated with improved neurogenesis and cognitive function [49,50], as well as increasing average birth weight [49]. Thus, it is probably worthwhile to explore the potential of olive oil supplementation in improving the birth weight of infants born to women who consume alcohol during pregnancy. However, unlike DHA, olive oil, when combined with alcohol in our study, did not increase the levels of pro-resolving lipid mediators in the brains of the alcohol-exposed pups (Figure 3). Existing evidence from the literature does not support any benefits of olive oil in counteracting alcohol’s inflammatory effects.

## 5. Limitations

There are some limitations in this study.

(1). This paper is essentially an exploratory study that demonstrates the response of pro-inflammatory and pro-resolving brain lipid mediators to alcohol, and the protective role of DHA. However, as an exploratory study, it is not comprehensive and many biomarkers of alcohol-induced degeneration—such as caspase 3 and 9 activation, DNA fragmentation, nuclear disruption, and pro-inflammatory cytokines like TNF-α, IL-1β, and TGF-β—are not included. Similarly, exploring the relationship with FASD phenotypes and brain pathology was beyond the initial aims of this study. Nonetheless, the research, especially its findings on the beneficial effects of DHA on pro-resolving brain lipid mediators, provides a foundation for future studies. These future investigations could offer valuable insight into the mechanisms of alcohol toxicity and potential treatment strategies in relation to prevention of the serious fetal and infant sequelae associated with maternal alcoholism. The Special Issue on “The Biological Impacts of Fetal Alcohol Exposure” aims to “bring together cutting-edge research from a range of disciplines to deepen our understanding of how prenatal alcohol exposure affects fetal development and long-term health outcomes”. Furthermore, despite decades of research, many questions remain about how alcohol disrupts critical biological processes during gestation, and how these disruptions result in the clinical features seen in Fetal Alcohol Spectrum Disorders (FASDs). We hope our study, though exploratory, aligns well with these goals.
(2). Adverse sex-specific responses to prenatal alcohol are known, such as in cognitive control [51], performance in differential tasks [52], and neuroimmune function [53]. Unfortunately, we were unable to determine the sex of the pups in this study, due to the difficulty we encountered in accurately measuring the anogenital distance in the extremely small pups. Molecular sex determination using Spry gene PCR determination on tissues has been suggested, and we can explore this option on frozen brain tissues of the pups, once funding becomes available.
(3). The present study of brain lipid mediators in the pups is only the first step in assessing the beneficial effects of DHA in mitigating the adverse impacts of alcohol on the fetal brain. Demonstrating an improvement in pups’ behavior is also necessary. We have an ongoing follow-up study using the same protocol which examines the anxiety behavior in rat pups at 6 weeks of postnatal age. Preliminary observations indicate that female rats perform better than male pups in the anxiety tests, which highlights the importance of sex.
(4). We could not determine sex and measure brain alcohol concentrations in the rat pups, nor blood alcohol concentrations in the dams, which are important factors that can directly reflect fetal brain exposure and response to alcohol. These factors will be determined in future studies.

## 6. Conclusions

We conclude that a high dose of 2.4 g/kg/day of alcohol significantly reduced the levels of pro-inflammatory and pro-resolving brain lipid mediators in rat pups prenatally exposed to alcohol. However, even at a high alcohol dose, the substantial decrease in pro-resolving brain lipid mediators was significantly reversed by DHA supplementation of the dams.

This study has potential implications for translational research in humans by using DHA to treat pregnant women who consume alcohol, aiming to prevent serious sequalae of alcohol injury such as FASD/FAS and intellectual disability in their offspring. To date, aside from abstaining from alcohol during pregnancy, there has been no effective intervention in humans to prevent these severe consequences of maternal alcoholism. DHA supplementation could represent a promising alternative to address this problem.

## Figures and Tables

**Table 1:** The mean birth weight (±SD) of rat pups at the delivery date of day 20 in the control, low-dose alcohol (LD), high-dose alcohol (HD), and high-dose alcohol + DHA (HD/DHA) treatment groups. Comparison was carried out by one-way ANOVA with Bonferroni correction. All dams were also given olive oil except the HD/DHA group.

					95% Confidence Interval	
Group	*N* (Dams)	*n* (Pups)	Mean Birth Weight (g)	SD	Lower Bound	Upper Bound
Control	6	48	3.06	0.25	2.80	3.32
LD	5	38	3.08	0.29	2.72	3.44
HD	5	40	4.10 *	0.25	3.79	4.41
HD/DHA	4	32	3.54 **	0.17	3.27	3.82

*N* = total number of dams per group (one dam in group 4 died from aspiration); *n* = total number of pups per treatment group (8 pups per dam, except 1 dam in the LD group who only had 6 pups); control = regular chow + olive oil; LD = low-dose alcohol (1.6 g/kg/day) + olive oil; HD = high-dose alcohol (2.4 g/kg/day) +olive oil; HD/DHA = high-dose alcohol (2.4 g/kg/day) + DHA (1250 mg/kg). One dam died from aspiration pneumonia; * *p* = 0.05 when compared to the control, LD, and HD/DHA groups; ** *p* = 0.05 when compared to the control, LD, and HD groups.

**Table 2:** Mean body weight (gms) ± SD of the dams on GDs 8 and 20 in the control, low-dose alcohol (LD), high-dose alcohol (HD), and high-dose alcohol + DHA (HD/DHA) treatment groups. Comparison was carried out by one-way ANOVA with Bonferroni correction. All dams were given olive oil except the HD/DHA group.

Weight (Grams) of the Dams on Day 8 and Day 20				
Day 8	N	Mean	Std. Deviation	Std. Error
Control	6	234.67	11.13	4.544
LD	5	251.0	17.916	8.012
HD	5	240.6	14.1	6.306
HD/DHA	4	234.8	12.377	5.535
				
Day 20				
Control	6	327.67	31.443	12.837
LD	5	343	45.591	20.389
HD	5	345.2	28.217	12.619
HD/DHA	4	321.2	39.915	17.85
ANOVA Day 8	*p* = 0.235			
ANOVA Day 20	*p* = 0.746			

*N* = total number of dams per group (one dam in the HD/DHA group died from aspiration); control = rat chow + olive oil; LD = low-dose alcohol (1.6 g/kg/day) + olive oil; HD = high-dose alcohol (2.4 g/kg/day) +olive oil; HD/DHA = high-dose alcohol (2.4 g/kg/day) + DHA (1250 mg/kg). One dam died from aspiration pneumonia.

**Table 3:** Mean placental weight (±SD) in the control, low-dose alcohol (LD), high-dose alcohol (HD), and high-dose alcohol + DHA (HD/DHA) groups. Comparison of means was carried out by one-way ANOVA with post hoc Duncan’s test.

Treatment Group	*N*	*n*	Mean Placental Weight (g)	SD	95% Confidence Interval	
					Lower Bound	Upper Bound
Control	6	48	0.49	0.08	0.46	0.51
LD	5	38	0.59	0.12	0.55	0.63
HD	5	40	0.57	0.07	0.55	0.59
HD/DHA	4	32	0.58	0.10	0.55	0.61

*N* = total number of dams per treatment group; *n* = total number of pups per treatment group (8 pups per dam except one dam in LD with 6 pups); control = rat chow + olive oil; LD = low-dose alcohol (1.6 g/kg/day) + olive oil (1 dam had only 6 pups); HD = high-dose alcohol (2.4 g/kg/day) +olive oil; HD/DHA = high-dose alcohol (2.4 g/kg/day) + DHA (1250 mg/kg). One dam died from aspiration pneumonia; *p* < 0.001 by one-way ANOVA.

**Table 4:** Duncan’s post hoc statistical test.

Duncan’s Test ^a,b^			
Group	*N*	Subset for Alpha = 0.05	
		1	2
Control	48	0.485	
High dose	40		0.568
DHA/alcohol	32		0.581
Low dose	38		0.592
Sig.		1.000	0.275

Means for groups in homogeneous subsets are displayed. ^a^ Uses a harmonic mean sample size of 38.685. ^b^ The group sizes are unequal. The harmonic mean of the group sizes is used. Type I error levels are not guaranteed.

## Data Availability

The datasets presented in this article are not readily available because the results of the paper are not published elsewhere and are not linked to archived database which can be shared.

## Supplementary Materials

The following supporting information can be downloaded at: https://www.mdpi.com/article/10.3390/life15101530/s1, Supplementary Materials S1. DHASCO oil composition.

## Abbreviations

The following abbreviations were used in this manuscript:

FASD	Fetal alcohol spectrum disorder
FAS	Fetal alcohol syndrome
PILMs	Pro-inflammatory lipid mediators
LTB4	Leukotriene B4
PGE2	ProstaglandinE2
PGF2	Prostaglandin F2α
TXB2	Thromboxane B2
PRLMs	Pro-resolving lipid mediators
LXA5	Lipoxin A5
4-HDoHE	4-Hydroxydocosahexaenoic acid
17-HDoHE	17-Hydroxydocosahexaenoic acid
MaR1n-3 DPA	7R,14S-dihydroxy-docosapentaenoic acid
PUFA	Polyunsaturated fatty acid
