# Supplementary material for: Docosahexaenoic Acid Increases the Pro-Resolving Brain Lipid Mediators of Inflammation in Rat Pups Prenatally Exposed to Alcohol
Source: Life (Basel). 2025 Sep 29;15(10):1530. doi: 10.3390/life15101530 (PMC12564984; doi:10.3390/life15101530)
Supplement: Supplementary file 1 [file life-15-01530-s001.zip › life-3802160-supplementary.pdf]

Supplement S1

1. DHASCO oil composition

DHASCO™

CERTIFICATE OF ANALYSIS

DSM

Product code	: 5013658005
Lot No.	: VY00241715
Analysis No.	: 77015511

Test	Result	Limits / Specifications	Dimension / Units
APPEARANCE	Corresponds	CLEAR FREE FLOWING LIQUID AT 40°C	
COLOR	Corresponds	LIGHT YELLOW TO DARK ORANGE	
AROMA	Corresponds	CHARACTERISTIC	
DOCOSAHEXAENOIC ACID	395	380 – 420	mg/g
DOCOSAHEXAENOIC ACID	42.5	40.0 – 45.0	%
PEROXIDE VALUE	< 0.1	Max. 5.0	meq/kg
ANISIDINE VALUE	7	Max. 20	
FREE FATTY ACID	0.1	Max. 0.4	%
UNSAPONIFIABLE MATTER	1.7	Max. 3.5	%
INSOLUBLE IMPURITIES	< 0.01	Max. 0.1	%
MOISTURE & VOLATILES	< 0.01	Max. 0.1	%
TRANS FATTY ACIDS	< 1	Max. 1	%
ELEMENTAL COMPOSITION			
Arsenic	Corresponds*	Max. 0.1	ppm
Cadmium	Corresponds*	Max. 0.1	ppm
Chromium	Corresponds*	Max. 0.2	ppm
Copper	Corresponds*	Max. 0.1	ppm
Iron	Corresponds*	Max. 0.2	ppm
Lead	Corresponds*	Max. 0.1	ppm
Manganese	Corresponds*	Max. 0.04	ppm
Mercury	Corresponds*	Max. 0.04	ppm
Molybdenum	Corresponds*	Max. 0.2	ppm
Nickel	Corresponds*	Max. 0.2	ppm
Phosphorus	Corresponds*	Max. 10	ppm
Silicon	Corresponds*	Max. 500	ppm
Sulfur	Corresponds*	Max. 100	ppm
FATTY ACID PROFILE			
8:0	0.3		%
9:0	< 0.1		%
10:0	1.2	Max. 5.0	%
11:0	< 0.1		%
12:0	5.1	Max. 15.0	%
13:0	< 0.1		%
14:0	12.9	Max. 25.0	%
14:1	0.3		%
16:0	11.3	Max. 20.0	%
16:1	2.4	Max. 10.0	%

DHASCO™

CERTIFICATE OF ANALYSIS

Product code : 5013658005
Lot No. : VY00241715
Analysis No. : 77015511

Test	Result	Limits / Specifications	Dimension / Units
17:0	< 0.1		%
17:1	< 0.1		%
18:0	0.8	Max. 5.0	%
18:1n-9	21.7	Max. 40.0	%
18:1n-7	0.1		%
18:2n-6	0.6	Max. 5.0	%
18:3n-3	< 0.1		%
20:0	< 0.1		%
20:1n-9	< 0.1		%
20:2n-6	< 0.1		%
20:3n-6	< 0.1		%
20:4n-6	< 0.1		%
20:5n-3 EPA	< 0.1	Max. 0.1	%
22:0	0.2		%
22:5n-3	0.5		%
22:6n-3 DHA	42.5	40.0 – 45.0	%
24:0	< 0.1		%
24:1	< 0.1	Max. 5.0	%
Others	< 0.1	Max. 5.0	%

*) checked at regular intervals
DSM endorses that this product meets the listed specifications through the 'best use before' date when the material is stored at the recommended temperature in the original, unopened container. Beyond the 'best use before' date, we recommend the quality of the product be confirmed by retesting the Certificate of Analysis parameters.

For questions regarding this Certificate of Analysis, please contact Customer Service.
Toll free (US Only) 1-800-662-6339 • International +1 (410) 740-0081

Released By:

Kimberly E Ketcham

Kim Ketcham - Senior Manager, Quality
